# Understanding sustainable purchase intention of smartphone users interface: Evidence from China

**DOI:** 10.3389/fpsyg.2023.1122801

**Published:** 2023-03-15

**Authors:** Lara F. Horani, Liangdong Dong

**Affiliations:** ^1^School of Economics and Management, Northwest University, Xi’an, China; ^2^Department of Business Administration, Northwest University, Xi’an, China

**Keywords:** purchase intention, sustainable design, sustainable requirements, traditional requirements, sustainable perceived value, price sensitivity

## Abstract

In recent decades, the fast development of smartphones has resulted in an enormous mass of e-waste besides a carbon footprint increase. In the face of serious environmental concerns, the manufacture and disposal of smartphones have become a primary customer concern. Environmental concerns are becoming a decisive factor when it comes to purchasing a product. Manufacturers have shifted their focus to product design with sustainable requirements in response to these new customer requirements. With all of the affordable technology manufacturers now may consider customer-sustainable requirements. This research aims to examine the relationship between traditional customer requirements, sustainable customer requirements, and sustainable purchase intention for smartphones in China, as well as the mediation effect of sustainable perceived value and the moderation effect of price sensitivity. Customers’ preferences are determined by using an online questionnaire. This research proposed an advanced sustainable purchase intention model by conducting an empirical analysis of the data gathered from 379 questionnaires. To gain a competitive advantage, companies should concentrate on meeting traditional and sustainable requirements more than the product price, according to the findings of the research. And contributes to the segmentation of the eco-friendly smartphone market.

## 1. Introduction

Rapid advances in technology in the 21st century have greatly impacted human lifestyles with a bevy of devices, technologies, and systems being integrated into humans life-changing the very fabric of society to an unprecedented extent. Asian countries in particular, due to fast-paced growth have been quick to adopt new technologies and incorporate them into their daily lives. Smartphones in particular have been immensely popular, integrating and transforming every aspect of life in Asian societies, ranging from being used for everything from personal to business activities in Asian countries with some level of industrialization (Wei et al., 2018). Gartner carried out surveys and found that, in the first quarter of 2021, smartphone sales globally exceeded 1.5 billion units, hitting a growth rate of 11.4% compared to last year’s data (Gartner, 2021). China has remained the world’s largest smartphone users since. In 2021 smartphone users in China reached over 911.92 million, accounting for about 66% percent of the total population in China (Statista, 2021).

Personal electronic devices’ rise and monumental growth have also seen a corresponding increase in humanity’s already formidable carbon footprint. Additionally, the life cycle of smartphones is limited due to advancements in functionality and features, as well as changes in people’s preferences. Due to shorter product cycles, phones get replaced consistently, and discarded smartphones become hazardous e-waste, contributing to environmental degradation as well as detrimental to human health (Kempen and Betzler, 2021). The United Nations “2020 Global E-waste Monitoring Report” stated that a staggering 53.6 million metric tons of e-waste were accumulated all around the world in 2019, up 21 percent in just 5 years – the highest increase among all types of other waste, with recycling tackling only 17.4% of it. The other 82.6% either ends up in landfills, is incinerated, resold, or repurposed through inappropriate channels and methods. Asia is the largest producer of e-waste. China ranked first, in terms of countries, then Japan and India in e-waste production, and this has become an urgent threat to China’s sustainable development. In 2020, over 2.7 million tons of e-waste were generated with the prediction for 2030 at an exponentially higher 81 million tons (GEM, 2018, 2020). Lead, lithium, mercury, and cadmium are among the highly processed and nearly inseparably amalgamated elements found in e-waste, which, if not handled properly will be the cause of substantial environmental degradation as well as a threat to human health (Tu et al., 2018).

The central focus of sustainable design is to design and produce products in a manner that is relatively more environmentally friendly while also being sensitive to the expectations of consumers and market sentiment. Product design has changed significantly from being concerned with traditional customer requirements like performance, shape, and easy-to-use, to sustainability considerations (social, economic, and environmental requirements) in the design process. The concept of sustainability in a product’s design philosophy is to balance environmental, economic, and social elements in the design of products and services. Indeed, sustainable product design is closely linked to the concept of sustainable Purchase Intention to minimize negative impacts and maximize positive impacts (Wang and Hsu, 2019).

In response, sustainable Purchase Intention is rapidly getting attention and traction, as a manifestation of the desire for people to pursue healthy lifestyles and choices with less destructive outcomes for the environment. Increasing public awareness of sustainability is leading to a corresponding rise in consumer purchasing decisions perceived as responsible. Environmental concerns have led to the identification of a new segment of consumers who care about the environment which is reflected in their purchasing decisions (Wang et al., 2021). Customer behavior is shifting toward awareness of environmental effects, to the point that some are even ready to pay more for sustainable product design products where effort has been made to reduce detrimental environmental impact (Tu et al., 2018). As an outcome, green development and sustainable purchasing have become trending issues of the time, Purchase Intention has been studied by several academics. Despite the fact that there have been major studies on this topic, there are significant gaps in the academic literature that need to be filled.

Research scholars have increasingly focused their attention on sustainable behavior with an emphasis on demographic variables through which the behavior of people towards sustainable Purchase Intention can be influenced (Al Mamun et al., 2018), and show that there are significant differences in the sustainable behavior of different consumers characteristics but other scholars believe that considering the demographic is not enough to reach a meaningful valuable conclusion, this has opened new perspectives on Purchase Intention. Therefore Scholars interested in the psychological variables of sustainable behavior have begun to explore consumers’ perceived values (Tu et al., 2018; Wang and Hsu, 2019). Consumers have strong opinions regarding different aspects of Smartphones revolving around connotations, information, and services. Additionally, functional, social, and emotional values also have an impact on the relationship between sustainable requirements, traditional requirements, and purchase intention, with such relationships expected to lead to more consumer-aware design (Wang and Hsu, 2019). Besides the influence of demographic variables on customer purchase intentions, this study attempts to examine how perceived value influences smartphone purchases with sustainability requirements when sustainability is highly valued.

Besides the demographic variables of consumers, and psychological variables price sensitivity is also closely involved in smartphone purchasing, Much of the research regards price sensitivity as a direct or indirect antecedent of sustainable customer behavior (Ghali-Zinoubi and Toukabri, 2019), and existing research has mainly focused on the correlation between environmentalism, green consumption, price sensitivity, and environmental concerns (Yue et al., 2020) but no study explores its moderating role on the relationship between purchase intention and sustainable and traditional requirements.

Many smartphone producers have realized that sustainable customer requirements will attract consumers’ attention and may affect their purchasing decisions, recent research has focused on the effect of environmental protection awareness on green customer behavior (Cerri et al., 2018). Other research has focused on smartphone consumers’ perceived values (Tu et al., 2018). And some research has explored the impact of just traditional requirements of smartphones on sustainable perceived value and purchase intention (Wang and Hsu, 2019). However, there is little to no previous research that explores the relationship between sustainable requirements, traditional requirements, perceived value, and purchase intention, also neglecting the impact of product prices which also exerts influence on consumers’ smartphone purchasing decisions. As a result, additional sustainable smartphone purchasing decision studies are needed.

We are still left with the questions of the process by which consumers consider sustainable requirements of the products during their purchase and consumption; how important it is for them to adopt sustainable requirements to product design, and how can we measure and compare the effect of sustainable requirements and the traditional ones on sustainable purchase intention? Accordingly, the aim of this research is to investigate the relationship between sustainable requirements, traditional requirements, and sustainable purchase intention. It takes into account the traditional requirements that affect the product design and compares them with the relevant sustainable requirements and impacts on sustainable purchase intentions, and the sustainable perceived value effect as a mediator and price sensitivity as a moderator, and even more so when they are also concerned about the environment.

Since the research works conducted on the relationship between sustainable customer requirements and purchase intention are limited, sustainable and traditional customer requirements influencers and their impact on sustainable consumers’ purchasing decision need to be explored further. For that one of the most significant distinctions of this work is to bridge the gap in the literature, it incorporates variables like sustainable requirements, traditional requirements, sustainable perceived value, and price sensitivity to the purchase intention model, additionally, this research proposes an advanced sustainable purchase intention model. This model examines the relationship between traditional customer requirements, sustainable customer requirements, and sustainable purchase intention for smartphones, as well as the mediation effect of sustainable perceived value and the moderation effect of price sensitivity. As compared to the previous customer purchasing model, which mainly considers the influence of traditional requirements on the customer purchasing decision.

While this study contributes to the literature on consumers’ purchasing intention, also it is to assist businesses in effectively targeting different customers’ requirements through segmentation analysis to promote marketing strategies that maximize profits while also satisfying customer needs for products with the least negative environmental impact. Regarding the intricacy and variety of variables that could influence sustainable purchase intentions, smartphones in china were selected for this research as a case study in which excellent representations of fast-changing, mass-market products.

Smartphones were selected for this case study which excellent representation of a fast-changings-market product. While the tech industry’s rapid expansion of smartphones has changed and eased customers’ lives, their production has created environmental issues and a large amount of e-west. It would be prudent for China to develop varied green specifications. The added cost of relatively environmentally safer products would be passed on to the consumers (Tu et al., 2018).

## 2. Literature review and hypotheses

This section proposes a review of the related literature on sustainable purchasing intention, as well as the hypotheses and their consideration that are used in the research model.

### 2.1. Sustainable purchase intention model and hypotheses development

The intention of a customer to buy a product is classified as a purchase intention. It can also be considered as a customer priority for a particular product while observing consumer purchasing behavior (Chen and Lin, 2019). The intention to purchase a product giving importance and preference to environmental considerations has been referred to as green purchase intention. Chen (2016) is of the opinion that green perceived value has a significant positive influence on sustainable purchase intention and eco-friendly purchasing is one part of sustainable behavior consumption (Chen, 2016).

There has been considerable research exploring the differentiation of different consumer classes and levels when it comes to identifying eco-friendly consumers using market segmentation approaches. Gender, age, education, family size, and income differences, have all been shown to have an essential influence on green consumption behavior in previous studies (Chekima et al., 2016), however, other scholars are of the opinion that analyzing the correlation between demographic variables and sustainable purchasing cannot conclusively prove the correlation (Sreen et al., 2018). Other avenues of research have explored the psychological mechanisms of consumers’ sustainable purchasing behavior based on classical interpretations of consumer behavior. To this end, the introduction of new psychological variables like “environmental knowledge,” “perceived green value,” and “perceived self-identification” was designated for the purpose of expanding upon the theory to encompass, analyze, and predict the effectiveness of green consumption behavior (Zhang et al., 2019; Yue et al., 2020). Furthermore, consumers with lower price sensitivity are more inclined to pay more, according to research by Hsu et al. (2017) price sensitivity was found to be a significant factor affecting purchase intentions (Hsu et al., 2017).

Moreover, the possible influence that price sensitivity and sustainable perceived value (in a Chinese context, specifically) on sustainable requirements, traditional requirements, and sustainable purchase intention has not been thoroughly investigated yet.

Following the purpose of this study. to examine the relationship between traditional customer requirements, sustainable customer requirements, and sustainable purchase intention for smartphones in China. Therefore, this research proposes a developed model based on Several theories and models on sustainable purchase intention: the sustainable customer behavior model that investigates the effect of demographic variables and product price on sustainable customer behavior (Huang et al., 2014; Lara, 2020). And also the theoretical conceptual models from an article titled “Does Sustainable Perceived Value Play a Key Role in the Purchase Intention Driven by Product Aesthetics? Taking smart watch as an Example” examines the impact of traditional requirements on sustainable perceived value and purchase intention (Wang and Hsu, 2019).

### 2.2. Sustainable customer requirements of electronic device design and sustainable purchase intention

Rapid economic growth in developing nations, China, in particular, has led to too much extremist natural resources consumption and accelerated environmental degradation (Li et al., 2019). Sustainable purchasing is seen as behavior that is environmentally responsible by supporting protecting nature and the environment and has stimulated the interest of businesses and consumers recently. Purchasing sustainable products for daily consumption is perceived to be an efficient way to deal with environmental issues (Yue et al., 2020).

Authoritative guidelines for ensuring sustainable growth of the electronics industry undergoing a phase of explosive growth were set by the International Electronics Manufacturing Initiative in 2005 and sought to specify future research, expansion, and functional requirement. Five key areas were to be focused upon: design, energy, recycling, materials, and sustainability (Tu et al., 2018).

Sustainability has been the focus of much academic concern. Paiano et al. (2013) take into consideration how the other users’ behavioral types would impact the smartphones, sustainability. Some academics have attention to the sustainability of the smartphone business model and developed a sustainable smartphone business model by integrating design, modularity in products, and the systems of product and service (Schneider et al., 2018). The rationale behind supporting this approach was to reduce the impact of smartphone production on the environment during the manufacturing phase, during the life cycle of a smartphone, this considers the majority of the emissions.

Changes in consumer requirements as a result of environmental concerns could affect the design and product time to the market. There is consensus in the industry now on obtaining sustainable requirements in the products along with traditional product requirements (Koçak et al., 2015; Alli et al., 2019).

Environmental is an important term in new product development because the whole life cycle of a product takes into account environmental requirements at all phases which would cause the least amount of environmental effect through the product’s life cycle, whereas eco-design considers environmental elements and economic elements during all product design phases. Those elements according to the World Business Council for Sustainable Development (WBCSD) include: Reducing the material intensity, reducing the energy intensity, minimizing the emission of toxic materials, increasing recyclability, enhancing sustainable use of renewable resources, improving durability, and increments in service intensity of goods and service (Chen and Liu, 2003), lately the definitions of sustainable design are also incorporating notions such as signifying a better quality of life in the second generation, the elements of the product design have changed to involve economic, social, and environmental elements such as working conditions, health and safety, wages, child labor, gender equity and social benefits such as fair trade and a living wage as well as the environmental influence throughout a product’s entire life cycle. The sustainable design then starts involving requirements like reduction of the material usage, ease of capability to process and assemble, transport, reduction of energy usage, low cost, durability, reusability, safe to use, safe level of emissions, capability to store, easy to clean and disassemble (Romli et al., 2015; Alli et al., 2019). These consumer expectations reflect the economic, social, and environmental considerations besides traditional requirements such as speed, resolution, easy disassembly for repair, incorporation of new technologies, reliability, size, weight, shape, ease of use, safe, durability, and large memory (Hsu et al., 2012; Wang and Hsu, 2019).

A total of 23 customer requirements for smartphones were obtained from literature reviews, interviews, and questionnaires. These requirements are further categorized into four categories: performance, easy to use, structure design requirements, and Sustainable.

There is clear supporting evidence that customer requirements for smartphones influence sustainable purchase intention. Considering the preceding arguments, it is expected that both sustainable and traditional requirements have a significant relationship with sustainable purchase intention. Based on the above, this research proposes the following hypotheses:

*Hypothesis 1*: sustainable requirements have a positive relationship with sustainable purchase intention.

*Hypothesis 2*: traditional requirements have a positive relationship with sustainable purchase intention.

*Hypothesis 2a*: performance requirements have a positive relationship with sustainable purchase intention.

*Hypothesis 2b*: ease of use requirements have a positive relationship with sustainable purchase intention.

*Hypothesis 2c*: structure design requirements have a positive relationship with sustainable purchase intention.

### 2.3. Sustainable perceived value and sustainable purchase intention

During the 1990s, the importance of focusing on customer value has been recognized as a dominant marketing concept. In this research, the concept of sustainable perceived value was associated with green perceived value and ecological perceived value, both of which have been correlated to consumers’ environmental attitudes and sustainable purchasing in earlier studies (Chen, 2013). Sustainable Perceived Value is a way for customers to convey their thoughts and ideas, as well as the value displayed by purchasing eco-friendly items. This manner of the propagation of ecological resources seems to be the outcome of a complex construct of cognitive and emotional factors. A number of factors can impact goal-oriented behavior, such as functions, society, and emotional factors (Tu et al., 2018).

Several researchers have indicated that the driving factors behind purchasing behaviors include functional, social, and emotional values (Toufani et al., 2017; Wang and Hsu, 2019). Therefore in this research, the sustainable perceived value was divided into three categories: functional value, social value, and emotional value, as listed below.

Functional value: The term “functional” is related to a product’s ability to provide a variety of advantages and features to its users.

Social value: related to how buyers may assume that by purchasing eco-friendly electrical products, they may improve their social position.

Emotional value: related to forming emotional attachments between users and products, and the environment.

Recently, Quade and Leimstoll (2017) propose a method to determine the influence of perceived value on the productive business operations of any company by studying the perceived value of smartphones for small and medium companies (Quade and Leimstoll, 2017).

Accordingly, this study assumes that the impact of customer requirements of smartphones will go through Sustainable perceived value first before it reaches sustainable purchase intention. Hence, this research proposes the following hypotheses:

*Hypothesis 3*: sustainable perceived value mediates the relationship between sustainable requirements and sustainable purchase intention.

*Hypothesis 4*: sustainable perceived value mediates the relationship between traditional requirements and sustainable purchase intention.

*Hypothesis 4a*: sustainable perceived value mediates the relationship between performance requirements and sustainable purchase intention.

*Hypothesis 4b*: sustainable perceived value mediates the relationship between easy-to-use requirements and sustainable purchase intention.

*Hypothesis 4c*: sustainable perceived value mediates the relationship between structure requirements and sustainable purchase intention.

### 2.4. Price sensitivity and sustainable purchase intention

Price sensitivity is the amount of user responsiveness to price changes as well as price variations between products (Ghali-Zinoubi and Toukabri, 2019). A lot of studies considered price sensitivity as an explicit or implicit predictor of whether or not customers would buy an eco-friendly product (Yue et al., 2020), but there is a dearth of research exploring its impact as a mediator between consumers’ consumption requirements and sustainable purchase intentions. Even in a situation where consumers expressed a desire to support eco-friendly products, it might not reflect in their actions as the price of such products with sustainable requirements is usually more than that of products with traditional requirements. Researchers discovered that price sensitivity played an essential role in purchase intentions, and often it would be seen that consumers with low price sensitivity would go for products such as electric cars (Hsu et al., 2017).

Based on data obtained from previous studies, it can thus be asserted that price-sensitive consumers have a lower likelihood of acting upon their environmental concerns which would lead to them having less impact on sustainable purchase intention. Therefore, this leads us to more hypotheses as well:

*Hypothesis 5*: The price moderates the relationship between sustainable requirements and sustainable purchase intention. This relationship is stronger when the price is higher than when it is lower.

*Hypothesis 6*: The price moderates the relationship between traditional requirements and sustainable purchase intention. This relationship is stronger when the price is higher than when it is lower.

### 2.5. The conceptual model

**Figure 1:**
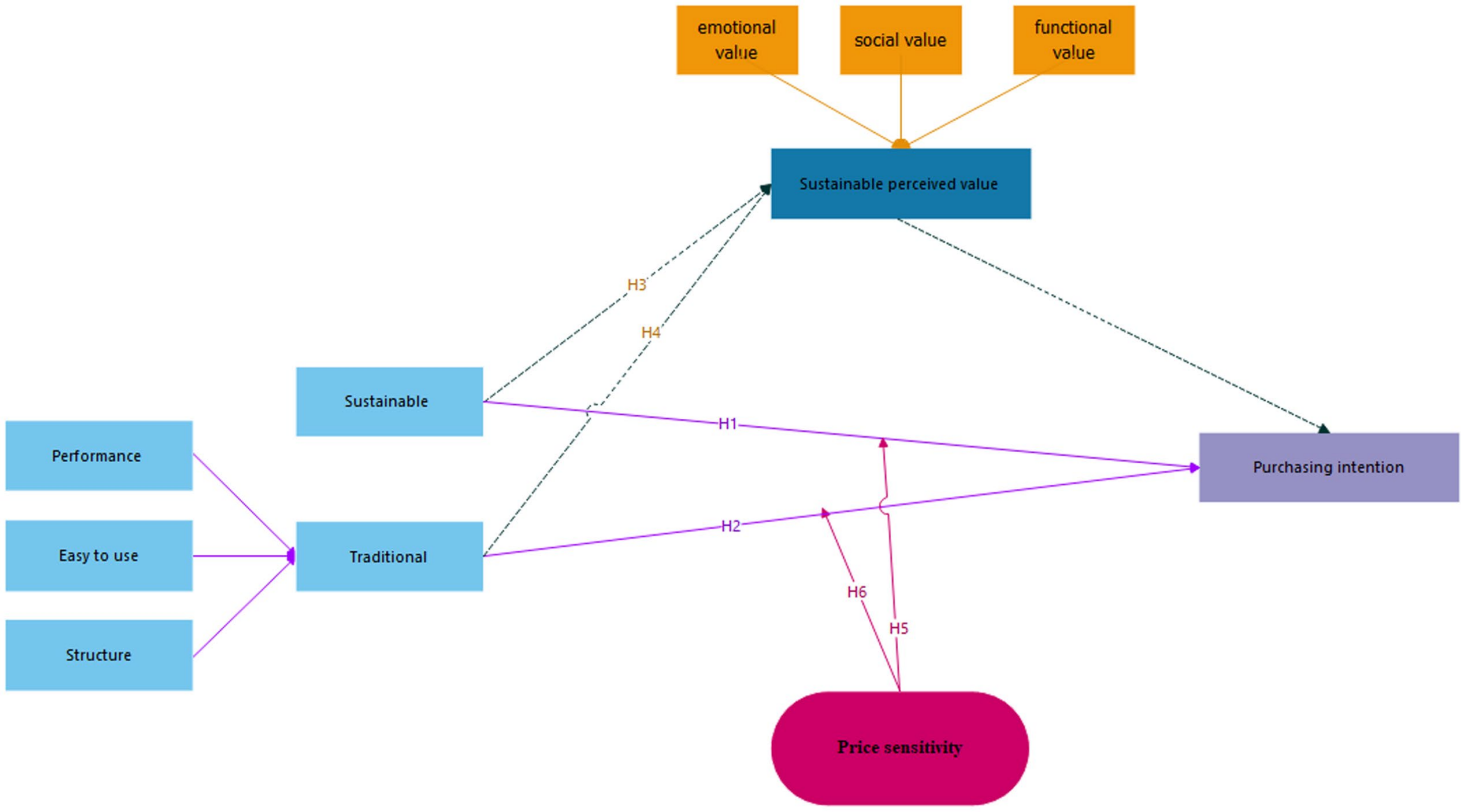
Sustainable purchase intention model.

Based on the above in the research literature review, the sustainable requirements and traditional requirements affect product design and have an impact on sustainable purchase intention, while the perceived sustainable value role as mediator and price sensitivity was a moderator. Figure 1 shows the research model.

## 3. Method of analysis and process

The process to measure the relationship between traditional customer requirements, sustainable customer requirements, and sustainable purchase intention for smartphones and to explore whether this relationship is moderated by price, as well as the mediation effect of sustainable perceived value is based on two phases: collecting the data in the first phase, and then analyzing it in the second. Questionnaires and interviews were used for the first phase of the data collection process. In the second phase, the SPSS (Version 23) program was used for the data analysis. The following are the analytical steps:

The first step is factor analysis, to examine the reliability and validity of all research variables, this study used Confirmatory factor analysis (CFA). The validity of the data was examined through the usage of KMO and Bartlett tests to verify sample size sufficiency and Cronbach’s α coefficient of the research variables was used to test reliability. Then correlation analysis was applied to examine the correlation between research variables.

Variance analysis in the second step, the T-test (T-test) and One-way ANOVA were applied to examine the score variations between the demographic variables on the research variables (Tu et al., 2018). To see whether there are any variations in scoring between genders and marital status on the research variables a t-test was carried out to determine the results, One-way ANOVA was implemented to examine the score variations between education level, age, and income on the research variables.

The third step tests the hypotheses by (1) analyzing the direct effect. A correlation in SPSS was performed to show the correlations between the research variables and determine the direction and significance of their relationships. Then regression analysis in SPSS (Version 23) was used to test the connection between sustainable requirements and purchase intention then traditional requirements and purchase intention, (2) examine the mediating effect of sustainable perceived value, to investigate if there is a link between sustainable requirements and sustainable purchase intention by sustainable perceived value, we used regression analysis in SPSS (Version 23), then used a Sobel test. The Sobel test is a method to test the significance of the mediation effect, which is basically a customized t-test that detects if the reduction in the independent variable’s influence after incorporating the mediator in the model is a significant reduction, and therefore whether the mediation effect is statistically significant (Wang and Hsu, 2019; Lara, 2020; Yue et al., 2020), (3) examine the moderating effect of price sensitivity, the PROCESS macro for SPSS was used to investigate the moderating effects of price sensitivity in this research by using Hayes’ moderated mediation method (Lara, 2020; Yue et al., 2020).

### 3.1. Measurement of variables

For the purpose of testing the proposed hypothesis, a qualitative and a quantitative cross-sectional survey design will be undertaken by this research. Due to the study having a cross-sectional design, the priorities and the assessment of the sample of customers on smartphones are gathered at a single point in time. To collect the relevant data, an online survey was carried out. Individuals are the object of analysis in this study.

There are two main components to the questionnaire. The demographic variables, or data collected on respondents’ socio-demographic variables, are presented in the first part (gender, age, marital status, educational levels, and monthly income). The second part examines five research variables; technical customer requirements, sustainable customer requirements price sensitivity, sustainable perceived value, and sustainable purchase intention. With respect to smartphones, the measurement of the questionnaire items in this research is modified from prior studies in order to evaluate the components in this research’s suggested research model Responses were recorded using a 5-point Likert scale extending from strongly disagree to strongly agree.

The smartphones customer requirements data was formed based on a review of the literature (Chen and Liu, 2003; Koçak et al., 2015; Alli et al., 2019; Wang and Hsu, 2019), and interviews, are divided into four aspects (sustainable, performance, ease of use, and structure design) and consists of 22 items (see Appendix A for the measurement items). The elements of the sustainable perceived values which are divided into three aspects (functional value, social value, and emotional value) were adapted from Koller et al. (2011) and consist of 6 items (see Appendix A for the measurement items). The measurement of purchase intention was made with two items scale adapted from Dehghani and Kim (2019). And According to Sinha and Batra (1999), a three-item scale was used to measure price sensitivity (see Appendix A for the measurement items).

### 3.2. Data collection and the sample

Consumers were presented with a formed questionnaire through the mini-program online survey platform that was built into the widely used WeChat app. An estimate in 2018 put the active daily users of WeChat at 350 million across China and the world, in this study, the survey was carried out on a sample of people of various ages, education levels, marital statuses, and income levels. In order to ensure an acceptable number of responses an online questionnaire survey has been adopted by using the WeChat app to distribute the questionnaires to various cities in China and just enabling participants who purchased smartphones to be eligible to participate in the survey. We sent 850 questionnaires and 541 were received, out of 541 questionnaires, 379 of the participants were able to qualify for the valid data who correctly completed the survey questions, and made up the representative sample. 379 were adequate for quite a high response rate of 70% during 5 months.

According to Boomsma and Hoogland (2001), researchers required at least 200 participants to apply structural equation modeling, therefore if the sample is more than 200 participants it is quite an efficient sample, therefore this study relatively has high reliability, and fulfills the condition of the sample size of more than 200, to complete the upcoming step of the research procedure.

As you can see in Table 1 the largest percentage of the participants was male 58.6, 54.6% of participants were single, and 41.7% of participants were aged (20–30) years. 44.3% of the participants had a level of education higher than a bachelor’s degree. The majority of participants (55.4%) reported their average monthly income to be between (1000–4,000) RMB, and 32.2% of the participants reported their average monthly income was above 6,000.

**Table 1:** Respondent of demographic variables (*N* = 379).

**Variables**	**Classification**	**Percent**
**Gender**	males	58.6
	females	41.4
**Marital status**	singles	54.6
	married	45.4
**Educational level**	lower than a bachelor’s degree	12.9
	bachelor’s degree	42.7
	more than a bachelor’s degree	44.3
**Age**	20–30	41.7
	31–40	38.3
	41–50	7.9
	51and more	12.1
**Income**	1,000–2000	32.2
	2000–4,000	23.2
	4,000–6,000	12.4
	6,000 and more	32.2

## 4. Analysis of empirical results

### 4.1. Factor analysis

#### 4.1.1. Reliability and validity analysis

This study used Confirmatory factor analysis (CFA) to test the reliability and validity of all research variables. The validity of the data was examined through the usage of KMO and Bartlett tests to verify sample size sufficiency. The KMO test for the five variables was 90.0%, and the Bartlett test results (were 5136.693, *p* < 0.001); the Bartlett test was also significant like KMO. Thus, it would be correct to conclude that sufficient data were gathered from the questionnaires to make it appropriate for moving forward with Factor Analysis.

Cronbach’s α coefficient of all research variables was used to test reliability. Generally, the lowest required value of Cronbach’s α coefficient is 0.70. Factor loadings were employed to measure convergent validity. Factor loadings and Alpha values were both greater than 0.5 and more than 0.7, respectively (Hair et al., 2006; Javed et al., 2022). As shown in Table 2 the Cronbach’s α coefficient of sustainable customer requirements was 0.807, traditional requirements were 0.811, the sustainable perceived value was 0.721, price sensitivity was 0.768, and purchase intention was 0.734, all of the measurements had a critical value of 0.70, showing that the measurements were reliable.

**Table 2:** Reliability and validity analysis of the research variables.

**Variables**	**Factor loading**	**Cronbach’s α**	**CR**	**AVE **	**✓ AVE**
1. Sustainable requirements	0.591	0.807	0.926	0.464	0.681
	0.639				
	0.671				
	0.831				
	0.578				
	0.751				
	0.570				
	0.683				
	0.768				
2. Traditional requirements		0.811	0.958	0.516	0.718
2.1 Performance requirements	0.739	0.812	0.933	0.527	0.726
	0.448				
	0.796				
	0.782				
	0.615				
	0.751				
	0.651				
	0.730				
2.2 Ease of use requirements	0.710	0.784	0.832	0.584	0.764
	0.739				
2.3 Design requirements	0.799	0.760	0.797	0.516	0.718
	0.773				
	0.734				
3. Perceived sustainable value		0.821	0.873	0.431	0.657
3.1 Functional value	0.552	0.756	0.872	0.652	0.807
	0.684				
3.2 Social value	0.500	0.779	0.874	0.658	0.811
	0.790				
3.3 Emotional value	0.640	0.793	0.902	0.714	0.845
	0.790				
4. Price sensitivity	0.855	0.768	0.879	0.601	0.775
	0.875				
	0.555				
5. Purchase intention	0.827	0.734	0.887	0.684	0.827
			0.827		

CMV = 28.7%, KMO and Bartlett’s Test = 90.0%, Bartlett’s Test (5136.693, *P* < 0.001).

The first variable value was 28.7%, below 50%. If the correlation coefficient of the comparative variables is more than 0.90, the CMV will be higher. Table 2 shows that the correlation coefficient’s maximum value was 0.637, which was significantly below 0.90, and the CMV has been within acceptable limits. As a result, this research’s CMV is relatively low.

#### 4.1.2. Descriptive statistics and correlation analysis

The means and standard deviations of the five variables are presented in Table 3, whereas Table 4 presents the correlation matrix, which shows the correlations between the research variables. According to correlation analysis, purchase intention was significantly positively correlated. With sustainable requirements (*r* = 0.470, *p* < 0.01), with traditional requirements (*r* = 0.366, *p* < 0.01), and with sustainable perceived value (*r* = 0.447, *p* < 0.01), while significantly negatively correlated with price sensitivity (*r* = −0.106, *p* < 0.05).

**Table 3:** Descriptive statistics.

**Variables**	**Mean**	**Std. deviation**
1. Sustainable requirements	4.283	0.740
2. Traditional requirements	4.196	0.676
2.1 Performance requirements	4.354	0.761
2.2 Easy-to-use requirements	4.075	1.007
2.3 Structure requirements	4.160	0.895
3. Sustainable perceived value	3.874	0.928
3.1 Functional value	4.346	0.949
3.2 Social value	3.471	1.304
3.3 Emotional value	3.806	1.252
4. Price sensitivity	3.651	1.205
5. Purchase intention	3.708	1.191

**Table 4:** Correlation analysis.

**Variables**	**1**	**2**	**2.1**	**2.2**	**2.3**	**3**	**3.1**	**3.2**	**3.3**	**4**	**5**
1.	1										
2.	0.773**	1									
2.1.	0.222**	0.363**	1								
2.2.	0.730**	0.664**	0.108*	1							
2.3.	0.637**	0.870**	0.697**	0.722**	1						
3.	0.517**	0.511**	0.357**	0.563**	0.614**	1					
3.1.	0.246**	0.369**	0.124*	0.365**	0.361**	0.368**	1				
3.2.	0.431**	0.409**	0.147**	0.566**	0.465**	0.477**	0.472**	1			
3.3.	0.485**	0.531**	0.246**	0.617**	0.587**	0.727**	0.806**	0.833**	1		
4.	−0.365**	−0.064	0.177**	−0.215**	−0.021	−0.132*	0.172**	−0.057	0.010	1	
5.	0.470**	0.366**	0.117*	0.494**	0.405**	0.447**	0.315**	0.424**	0.491**	−0.106*	1

* Correlation was significant at *p* < 0.05 (2-tailed).

** Correlation was significant at *p* < 0.01 (2-tailed).

### 4.2. Variance analysis

#### 4.2.1. One-way ANOVA analysis and *T*-test on the research variables

This research examined the score variations between the five socio-demographic variables (gender, married status, education level, age, and income) on the research variables which are identified in the earlier section. To see whether there are any variations in scoring between genders and marital status on the research variables a *t*-test was carried out to determine the results, One-way ANOVA was implemented to examine the score variations between education level, age, and income on the research variables.

The scores scaled by gender achieved a significant level for sustainable requirements, as shown in Table 5, as judged (value of *p* < 0.05). Males had a higher mean score than females, in favor of males with a mean of 4.346, and reached a significant level for easy-to-use requirements. Males had a higher mean score than females, in favor of males with a mean of 4.223. Regarding the other factors, the scores did not reach significance levels (value of *p* > 0.05), thus gender variations did not reveal significantly different perceived values for research variables.

**Table 5:** *T*-test of gender on research variables.

**Variables**	**Gender**	**No.**	**Mean**	**S.D.**	***t* value**	***P* value**
1.	Males	222	4.346	0.762	1.976	**0.049**
	Females	157	4.194	0.700		
2.	Males	222	4.224	0.722	0.934	0.351
	Females	157	4.158	0.606		
2.1.	Males	222	4.315	0.794	−1.201	0.230
	Females	157	4.410	0.710		
2.2.	Males	222	4.223	1.018	3.447	**0.001**
	Females	157	3.866	0.955		
2.3.	Males	222	4.134	0.945	−0.684	0.495
	Females	157	4.197	0.819		
3.	Males	222	3.841	0.998	−0.832	0.406
	Females	157	3.921	0.820		
3.1.	Males	222	4.349	0.948	0.084	0.933
	Females	157	4.341	0.954		
3.2.	Males	222	3.466	1.404	−0.084	0.933
	Females	157	3.478	1.154		
3.3.	Males	222	3.707	1.369	−1.834	0.067
	Females	157	3.946	1.054		
4.	Males	222	3.712	1.235	1.170	0.243
	Females	157	3.565	1.160		
5.	Males	222	3.662	1.278	−0.899	0.369
	Females	157	3.774	1.057		

With respect to the influence of marital status, the scores scaled by marital status achieved a significant level for performance requirements as judged (value of *p* < 0.05). Singles had a higher mean score than married, in favor of singles with a mean of 4.487, and reached a significance level for easy-to-use requirements as judged (value of *p* < 0.05). Married had a higher mean score than singles, in favor of males with a mean of 4.227. Regarding the research variables, the scores of all of the research variables did not reach significance levels (value of *p* > 0.05), As a result, there were no significant differences in perceived values for the research variables across respondents of various marital statuses (Table 6).

**Table 6:** *T*-test of marital status on research variables.

**Variables**	**Marital status**	**No.**	**Mean**	**S.D.**	***t* value**	***P* value**
1.	Singles	207	4.303	0.656	0.603	0.547
	Married	172	4.258	0.832		
2.	Singles	207	4.225	0.690	0.892	0.373
	Married	172	4.163	0.659		
2.1.	Singles	207	4.487	0.632	3.783	**0.000**
	Married	172	4.195	0.867		
2.2.	Singles	207	3.949	1.048	−2.693	**0.007**
	Married	172	4.227	0.936		
2.3.	Singles	207	4.238	0.854	1.874	0.062
	Married	172	4.066	0.935		
3.	Singles	207	3.892	0.901	0.411	0.681
	Married	172	3.853	0.962		
3.1.	Singles	207	4.355	0.965	0.212	0.832
	Married	172	4.334	0.934		
3.2.	Singles	207	3.531	1.273	0.989	0.323
	Married	172	3.398	1.341		
3.3.	Singles	207	3.79	1.239	−0.276	0.783
	Married	172	3.826	1.271		
4.	Singles	207	3.733	1.053	1.453	0.147
	Married	172	3.552	1.362		
5.	Singles	207	3.812	1.068	1.855	0.064
	Married	172	3.584	1.317		

In regards to respondent scores at different levels of education (lower than bachelor’s degree, bachelor’s degree, higher than bachelor’s degree), the scores sorted by education levels for easy-to-use requirements did not reach a significant level as judged (value of *p* > 0.05), while reaching significance levels for all other research variables (value of *p* < 0.05).

Table 7 shows the differences between education levels on sustainable requirements, respondents with education levels higher than bachelor’s (M = 4.55) had much more positive attention to sustainable requirements than respondents with other education levels. Respondent scores among different education levels related to traditional requirements compared with those with bachelor’s degrees and higher did not reach significance levels (value of *p* > 0.05), while the other categories did reach significance levels (value of *p* < 0.05) in favor of those with a higher level of education (M = 4.35). With respect to the scores of respondents with different education levels on sustainable perceived value for bachelor’s degree and higher did not reach significance levels (value of *p* > 0.05), while the other categories did reach significance levels (value of *p* < 0.05) in favor of higher than bachelor’s (M = 4.06). Scores of respondents with different education levels on the price sensitivity value for bachelor’s degree and higher did not reach significance levels (value of *p* > 0.05) while reaching significance levels (value of *p* < 0.05) for other categories in favor of lower than bachelor’s (M = 4.51); and the scores of respondents with different education levels on purchase intention for bachelor’s degree and higher than bachelor did not reach significance levels (value of *p* > 0.05) with other categories reaching significance levels (value of *p* < 0.05), in favor of higher than bachelor’s (M = 4.00).

**Table 7:** One-way ANOVA of education on research variables.

**Variables**	**Education**	**No.**	**Mean**	**S.D.**	**f value**	***P* value**
1.	1. lower than bachelor’s	49	3.175	0.688	102.29	**0.000**
	2. bachelor’s degree	162	4.337	0.613		
	3.more than bachelor’s	168	4.554	0.552		
2.	1. lower than bachelor’s	49	3.571	0.649	29.09	**0.000**
	2.bachelor’s degree	162	4.229	0.654		
	3.more than bachelor’s	168	4.347	0.603		
2.1.	1. lower than bachelor’s	49	3.270	0.963	82.939	**0.000**
	2.bachelor’s degree	162	4.461	0.620		
	3.more than bachelor’s	168	4.567	0.523		
2.2.	1. lower than bachelor’s	49	4.061	1.093	1.643	0.195
	2. bachelor’s degree	162	3.975	1.006		
	3.more than bachelor’s	168	4.176	0.978		
2.3.	1. lower than bachelor’s	49	3.381	0.861	24.086	**0.000**
	2. bachelor’s degree	162	4.251	0.847		
	3. more than bachelor’s	168	4.300	0.837		
3.	1. lower than bachelor’s	49	3.060	0.860	25.05	**0.000**
	2. bachelor’s degree	162	3.930	0.880		
	3. more than bachelor’s	168	4.060	0.880		
3.1.	1. lower than bachelor’s	49	3.582	1.165	20.781	**0.000**
	2. bachelor’s degree	162	4.401	0.874		
	3. more than bachelor’s	168	4.515	0.843		
3.2.	1. lower than bachelor’s	49	2.949	0.964	4.598	**0.011**
	2. bachelor’s degree	162	3.540	1.272		
	3. more than bachelor’s	168	3.557	1.390		
3.3.	1. lower than bachelor’s	49	2.663	1.092	28.982	**0.000**
	2. bachelor’s degree	162	3.843	1.238		
	3. more than bachelor’s	168	4.104	1.120		
4.	1. lower than bachelor’s	49	4.510	0.860	15.98	**0.000**
	2. bachelor’s degree	162	3.570	1.200		
	3. more than bachelor’s	168	3.460	1.200		
5.	1. lower than bachelor’s	49	2.520	0.880	34.88	**0.000**
	2. bachelor’s degree	162	3.770	1.190		
	3. more than bachelor’s	168	4.000	1.060		

As for respondents with different ages (20–30, 31–40, 41–50, and 51and more), the scores sorted by age for easy-to-use requirements did not reach a significance level, as judged (value of *p* > 0.05). While reaching significant levels for all other research variables (value of *p* < 0.05).

Table 8 shows that there are differences between the ages of sustainable requirements respondents for all categories (group) with (51 and more), in favor of other categories. There are differences between ages on traditional requirements respondents for all categories with (51 and more), in favor of other categories. Regarding the differences of respondents with ages on sustainable perceived value respondents for all categories with (51 and more), were in favor of other categories. There are differences between the ages of price sensitivity value respondents for all categories with (51 and more), in favor of (51 and more; M = 4.63), also for (20–30) with (41–50) in favor of (20–30; M = 3.70), and for (31–40) with (41–50) in favor of (31–40; M = 3.52). Finally, there are differences between the ages of purchase respondents for all categories (51 and more), in favor of other categories.

**Table 8:** One-way ANOVA of age on research variables.

**Variables**	**age**	**No.**	**Mean**	**S.D.**	**f value**	***P* value**
1.	20–30	158	4.32	0.65	48.634	**0.000**
	31–40	145	4.50	0.58		
	41–50	30	4.58	0.37		
	51 and above	46	3.26	0.83		
2.	20–30	158	4.32	0.64	17.507	**0.000**
	31–40	145	4.27	0.64		
	41–50	30	4.19	0.48		
	51 and above	46	3.57	0.71		
2.1.	20–30	158	4.550	0.615	86.758	**0.000**
	31–40	145	4.513	0.498		
	41–50	30	4.563	0.293		
	51 and above	46	3.046	0.838		
2.2.	20–30	158	4.089	0.991	2.057	0.106
	31–40	145	4.048	0.981		
	41–50	30	3.750	0.917		
	51 and above	46	4.326	1.151		
2.3.	20–30	158	4.308	0.863	17.286	**0.000**
	31–40	145	4.241	0.866		
	41–50	30	4.267	0.528		
	51 and above	46	3.326	0.853		
3.	20–30	158	3.99	0.87	17.473	**0.000**
	31–40	145	3.95	0.90		
	41–50	30	4.20	0.76		
	51 and above	46	3.02	0.87		
3.1.	20–30	158	4.453	0.817	13.251	**0.000**
	31–40	145	4.424	0.914		
	41–50	30	4.600	0.532		
	51 and above	46	3.565	1.289		
3.2.	20–30	158	3.573	1.311	4.667	**0.003**
	31–40	145	3.497	1.361		
	41–50	30	3.783	1.343		
	51 and above	46	2.837	0.830		
3.3.	20–30	158	3.956	1.219	17.254	**0.000**
	31–40	145	3.924	1.217		
	41–50	30	4.217	0.907		
	51 and above	46	2.652	1.059		
4.	20–30	158	3.70	1.01	23.189	**0.000**
	31–40	145	3.52	1.20		
	41–50	30	2.51	1.33		
	51 and above	46	4.63	0.97		
5.	20–30	158	3.90	1.01	24.205	**0.000**
	31–40	145	3.79	1.28		
	41–50	30	4.23	0.75		
	51 and above	46	2.46	0.92		

As for respondents with different incomes (1000–2000, 2000–4,000, 4,000–6,000, and 6,000 and more), they reached a significance level for sustainable requirements, performance requirements, structure requirements, sustainable perceived value, and price sensitivity, as judged (value of *p* < 0.05). The rest of the research variables did not reach significance levels (value of *p* > 0.05), As a result, there were no significant differences in perceived values for the rest of the research variables across responders of different income levels. Table 9 shows that there are different income on sustainable requirements for respondents (1000–2000) RMB with (4000–6,000) RMB, and also for (2000–4,000) RMB with (4000–6,000) RMB, in favor of (4000–6,000) RMB for both (M = 4.65). Regarding the differences of respondents with income on sustainable perceived value for (1000–2000) RMB with (4000–6,000) RMB, and also for (2000–4,000) RMB with (4000–6,000) RMB, in favor of (4000–6,000) RMB for both (M = 4.34). The differences of respondents with income on price sensitivity value (1000–2000) RMB with (4000–6,000) RMB in favor of (1000–2000) RMB (M = 3.77), also for (2000–4,000) RMB with (4000–6,000) RMB in favor of (2000–4,000) RMB for both (M = 4.09), and (2000–4,000) RMB with (6,000 and more) RMB, in favor of (2000–4,000) \ for both (M = 4.09).

**Table 9:** One-way ANOVA of income on research variables.

**Variables**	**Income**	**No.**	**Mean**	**S.D.**	**f value**	***P* value**
1.	1,000–2000	122	4.19	0.67	5.911	**0.001**
	2000–4,000	88	4.14	0.81		
	4,000–6,000	47	4.65	0.52		
	6,000 and more	122	4.33	0.78		
2.	1,000–2000	122	4.26	0.65	2.637	0.050
	2000–4,000	88	4.04	0.72		
	4,000–6,000	47	4.35	0.72		
	6,000 and more	122	4.19	0.64		
2.1.	1,000–2000	122	4.452	0.697	6.225	**0.000**
	2000–4,000	88	4.246	0.761		
	4,000–6,000	47	4.694	0.505		
	6,000 and more	122	4.204	0.851		
2.2.	1,000–2000	122	4.016	0.960	2.065	0.104
	2000–4,000	88	3.972	1.118		
	4,000–6,000	47	3.947	1.134		
	6,000 and more	122	4.258	0.898		
2.3.	1,000–2000	122	4.303	0.875	4.709	**0.003**
	2000–4,000	88	3.917	0.863		
	4,000–6,000	47	4.404	0.828		
	6,000 and more	122	4.098	0.921		
3.	1,000–2000	122	3.86	0.90	6.783	**0.000**
	2000–4,000	88	3.61	0.97		
	4,000–6,000	47	4.34	0.70		
	6,000 and more	122	3.90	0.94		
3.1.	1,000–2000	122	4.340	0.963	5.781	**0.001**
	2000–4,000	88	4.028	1.084		
	4,000–6,000	47	4.660	0.685		
	6,000 and more	122	4.459	0.861		
3.2.	1,000–2000	122	3.488	1.294	2.178	0.090
	2000–4,000	88	3.295	1.281		
	4,000–6,000	47	3.883	1.336		
	6,000 and more	122	3.422	1.300		
3.3.	1,000–2000	122	3.738	1.252	6.864	**0.000**
	2000–4,000	88	3.500	1.309		
	4,000–6,000	47	4.489	0.741		
	6,000 and more	122	3.832	1.279		
4.	1,000–2000	122	3.77	0.94	9.625	**0.000**
	2000–4,000	88	4.09	0.91		
	4,000–6,000	47	3.09	1.37		
	6,000 and more	122	3.43	1.42		
5.	1,000–2000	122	3.65	1.11	2.494	0.060
	2000–4,000	88	3.78	1.17		
	4,000–6,000	47	4.10	1.00		
	6,000 and more	122	3.57	1.32		

### 4.3. Hypotheses testing

#### 4.3.1. Analyzing the direct effect

Regression analysis in SPSS (Version 23) was used to test the connection between sustainable requirements and purchase intention. Table 10 presents the findings of this research’s main effect analysis. H_1_ expected that sustainable requirements have a positive relationship with sustainable purchase intention, which is supported (*β* = 0.756, value of *p* < 0.001). In addition, traditional requirements have a positive relationship with sustainable purchase intention, which is supported by H_2_ (*β* = 0.713, value of *p* < 0.001). H_2a_ predicted that performance requirements would have a positive relationship with sustainable purchase intention, which is supported (*β* = 0.488, value of *p* < 0.001), and H_2b_ also predicted that ease of use requirements would have a positive relationship with the sustainable purchase intention, which is supported (*β* = 0.138, value of *p* < 0.05), and design requirements were hypothesized to have a positive relationship with sustainable purchase intention, which is supported with H_2c_ (*β* = 0.773, value of *p* < 0.001).

**Table 10:** The results of the direct effect analysis.

**Hypothesis**	**The effect**	**Path coefficient**	**S.E.**	***t* value**	***P* value**	**Results**
H_1_	+	0.756	0.318	1.475	0.000**	H_1_ is supported
H_2_	+	0.713	0.083	8.595	0.000**	H_2_ is supported
H_2a_	+	0.488	0.064	7.640	0.000**	H_2a_ is supported
H_2b_	+	0.138	0.061	2.288	0.023*	H_2b_ is supported
H_2c_	+	0.773	0.070	11.033	0.000**	H_2c_ is supported

* Significant at the *p* < 0.05 (2-tailed).

** Significant at the *p* < 0.01 (2-tailed).

#### 4.3.2. Analysis of the mediating effect of sustainable perceived value

To investigate if there is a link between sustainable requirements and sustainable purchase intention by sustainable perceived value, we used regression analysis in SPSS (Version 23), then used a Sobel test. The Sobel test is a method to test the significance of the mediation effect, which is basically a customized *t*-test that detects if the reduction in the independent variable’s influence after incorporating the mediator in the model is a significant reduction, and therefore whether the mediation effect is statistically significant.

Table 11 shows the expected indirect effect of sustainable requirements on sustainable perceived value, which is supported (*β* = 0.608, S.E. = 0.056), and the direct effect of sustainable perceived value on purchase intention (*β* = 0.630, S.E. = 0.058), then followed by application of the Sobel test which is supported with H_3_ (S.E. = 0.049, value of *p* < 0.001), thus highlighting how sustainable perceived value mediates the relationship between sustainable requirements and sustainable purchase intention.

**Table 11:** The regression results of the mediation analysis.

**Hypothesis**	**Path**	**Effects**	**Path coefficient**	**S.E.**	***t* value**	***P* value**	**Results**
H_3_	SR-SPV-PI	Indirect Effect	0.608	0.056	10.772	0.000	H_3_ is supported
		Direct Effect	0.630	0.058	10.932	0.000	
		Total Effect		0.049	7.679	0.000	
H_4_	TR-SPV-PI	Indirect Effect	0.806	0.057	14.091	0.000	H_4_ is supported
		Direct Effect	0.630	0.058	10.932	0.000	
		Total Effect		0.059	8.614	0.000	
H_4a_	PR-SPV-PI	Indirect Effect	0.753	0.049	15.228	0.000	H_4a_ is supported
		Direct Effect	0.630	0.058	10.932	0.000	
		Total Effect		0.053	8.870	0.000	
H_4b_	US-SPV-PI	Indirect Effect	0.226	0.046	4.919	0.000	H_4b_ is supported
		Direct Effect	0.630	0.058	10.932	0.000	
		Total Effect		0.031	4.476	0.000	
H_4c_	STR-SPV-PI	Indirect Effect	0.551	0.045	12.167	0.000	H_4c_ is supported
		Direct Effect	0.630	0.058	10.932	0000	
		Total Effect		0.043	8.125	0.000	

Sustainable requirements (SR), purchase intention (PI), sustainable perceived value (SPV), traditional requirements (TR), performance requirements (PR), easy-to-use requirements (US), and structure requirements (STR).

In addition, sustainable perceived value mediates the relationship between traditional requirements and sustainable purchase intention which is supported by H_4_ (S.E. = 0.059, value of *p* < 0.001). H_4a_ predicted that sustainable perceived value mediates the relationship between performance requirements and sustainable purchase intention, which is supported (S.E. = 0.053, value of *p* < 0.001). H_4b_ predicted that sustainable perceived value mediates the relationship between easy-to-use requirements and sustainable purchase intention, which is supported (S.E. = 0.031, value of *p* < 0.001), and H_4c_ predicted that sustainable perceived value mediates the relationship between design requirements and sustainable purchase intention, which is supported (S.E. = 0.043, value of *p* < 0.001).

#### 4.3.3. Analysis of the moderating effect of price sensitivity

The PROCESS macro for SPSS was used to investigate the moderating effects of pricing by using Hayes’ moderated mediation method. Table 12 shows the results of the moderating effects of price sensitivity in this research. Sustainable requirements and price sensitivity interaction indicated that H5 is not supported since the moderating effects of price sensitivity between sustainable requirements and purchase intention were not significant [*p*-value (Interaction1) < 0.001, while value of *p* (Price) > 0.05].

**Table 12:** The regression results of the moderation analysis.

**Hypothesis**	**Model**	**Coefficient** β**’s**	**S.E.**	***t* Value**	***P* value**	**95% confidence interval for** β		**Results**
						**Upper**	**Lower**	
H_5_	Constant	1.108	0.404	2.739	0.006	0.869	3.229	H_5_ is not supported
	SR	0.627	0.087	7.217	0.000	0.147	0.667	
	Price	−0.034	0.116	−0.296	0.767	−0.263	0.194	
	Interaction1	0.193	0.060	3.229	0.001	0.153	0.628	
H_6_	Constant	1.046	0.359	2.915	0.004	0.697	2.618	H_6_ is supported
	TR	−0.330	0.112	−2.935	0.004	0.300	0.747	
	Price	0.670	0.083	8.099	0.000	−0.551	−0.109	
	Interaction2	0.117	0.055	2.131	0.034	0.018	0.455	

Sustainable requirements (SR) and traditional requirements (TR).

However, traditional requirements and price sensitivity interaction indicated that H6 is supported since the price sensitivity moderates the relationship between traditional requirements and purchase intention and influences the negative impact of traditional requirements on purchase intention [value of *p* (Interaction2) < 0.001, while value of *p* (Price) < 0.0].

## 5. Discussion

This research examines the impact of sustainable customer requirements on the Purchase Intention of Smartphones in China as well as the traditional requirements. Given that China has the most smartphone users in the world, this is a crucial area to concentrate on (Gartner, 2021). Additionally, due to shorter product cycles, phones get replaced consistently, and discarded smartphones become hazardous e-waste, contributing to environmental degradation as well as detrimental to human health. Based on the research hypotheses (H1) expected that sustainable requirements have a positive relationship with sustainable purchase intention. And (H2) traditional requirements have a positive relationship with sustainable purchase intention. Results revealed that Sustainable requirements showed a strong positive correlation with purchase intention, and were the strongest among all the other variables, especially against traditional requirements which were relatively weaker, this observation is consistent with the previous research (Wang and Hsu, 2019; Kempen and Betzler, 2021) and among traditional requirements of smartphones, the performance aspect held greater significance than easy-to-use and structure requirements. However, price played an important role in the purchase decision and has an impact on consumer requirements of smartphones. At the same time, this research examined the score variations between the five socio-demographic factors (gender, married status, education level, age, and income) on the research variables and the findings revealed that males with more than a bachelor’s degree, aged between 41 to 50, and earning a monthly income between 4,001 to 6,000 RMB had a significant influence on the sustainable requirements of smartphones. This result indicated that males with high education levels and aged between 41 to 50 were more knowledgeable and more concerned over environmental issues and sustainable design and had a correspondingly high income making them less sensitive toward prices, showing higher purchase intention toward sustainable smartphones such as recyclability, energy saving, no toxic material released, less waste, lower environmental impact, safe, information and data security, durability, and easy maintenance, even if those features made the products expensive. Based on previous research, females exhibited a higher level of environmental consciousness than males and a willingness to pay more for sustainable designs (Laroche et al., 2001; Tu et al., 2018). In this research, males had a significant influence on the sustainable requirements of smartphones, which may be related to the sample size, and the majority of the responders were males. Because gender influences sustainable requirements, smartphone manufacturers can design suitable strategies to satisfy target customer preferences.

Gender did not significantly affect traditional requirements and those aged between 20 to 30 had a significant influence on traditional requirements of smartphones. The results in this category indicated that young customers seemed to be less concerned about the environment and sustainable design and more into traditional requirements such as the reliability of smartphones, large memory, and high-resolution screens which they preferred over easy-to-access, shape, and size requirements to suit their needs more. And since they have a monthly income ranging between 2000 to 4,000 RMB, the price of products is given a lot of importance by them making them more sensitive to the prices of smartphones. This result might be explained by the fact that young who has low-income respondents deliberate the most when expending a restricted amount of funds.

Regarding H3, H4, and (H4a H4b H4c H4d) the results showed that all sustainable perceived value factors (social value, functional and emotional value) have a positive effect on purchase intention and mediate the relationship between sustainable requirements and purchase intention, and between traditional requirements and purchase intention.

Performance is a basic requirement of customers of smartphones, especially young customers who are more into traditional requirements which enhance the functional value for them when they make their purchasing decisions. Toufani et al. (2017) on the other hand suggest that social value influences purchase intention the most, while functional value has almost no impact. When consumers buy smartphones with sustainable requirements, they present their concern toward environmental protection, and people with greater social positions have more solid motivations at the same time they care to show their society how they are involved with protecting the environment and the planet. And consumers sometimes want to show their social level or their living standard because expensive or latest smartphones can be used to signify status. At the same time, the functional value affects their purchasing decisions too so they prefer the quality and requirements such as reliability, big memory, high resolution, and easy-to-access for the customers who try to keep updated with the latest technology.

Many researches indicated that emotional value has a significant influence on smartphone purchase intention (Toufani et al., 2017; Wang and Hsu, 2019). According to Chapman (2012), sustainability concerns may be handled by focusing on the product’s design lifecycle and linking it to people’s emotional needs. Consumers seeking to buy smartphones at the beginning might be attracted to the structure, but what is more important when they make the final purchasing decision is the value they get from the smartphone, this research examined the effect of sustained perceived value as a mediator in increasing purchase intention.

Smartphone producers currently are not playing any role in protecting the environment now have to consider the sustainable design and expand product lifetime by obtaining a design method focused on the product lifecycle. Since the durability of the product influences emotional value, it has a positive effect on purchase intention. To enhance sustainable design, designers should focus on increasing the lifespan of the product. Hsiao and Chen (2018) presented a comprehensive approach to enhancing sustainable design relationships by focusing on consumer requirements, values, and emotions.

In the relationship between traditional requirements and purchase intention, price sensitivity plays a negative moderator. That is, customers with low price sensitivity are more willing to buy smartphones than customers with high price sensitivity. On the other hand, the positive relationship between sustainable requirements and purchase intention is not moderated by price sensitivity, and H5 is not supported. This research proposes, backed by empirical data, that consumers who purchase sustainable design do not have price sensitivity, and display a willingness to acquire smartphones with sustainable requirements even if they are more expensive than competing products. They are influenced by environmental consciousness, which leads to sustainable purchase intention. The findings are consistent with numerous prior studies that have shown that price sensitivity has a negative impact (Stall-Meadows and Davey, 2013; Yue et al., 2020). For that manufacturers should shift customers’ attention and consideration toward the price of smartphones with sustainable requirements by highlighting the importance and value of sustainable design requirements.

## 6. Conclusion

The purpose of this research was to investigate the relationship between sustainable requirements, traditional requirements, and purchase intention, and how this relationship was mediated by sustainable perceived value and moderated by price sensitivity when customers purchase smartphones. The three main conclusions of this research are: First, the independent variables, which include sustainable requirements and traditional requirements, had a significant positive relationship with purchase intention. Second sustainable perceived value had a significant mediating effect on the relationship between the independent variables (sustainable requirements and traditional requirements) and purchase intention Third, price sensitivity did moderate the relationship between traditional requirements and purchase intention and it also reduced the negative effect of traditional requirements on purchase intention, while the moderating effect of price sensitivity on the relationship between sustainable requirements and purchase intention was not significant. Hence Companies that produce smartphones are recommended to use sustainable marketing to emphasize the hidden value of sustainable requirements with a green focus to help the consumers to understand the extra benefits and the added value of the spent money to contribute to protecting the environment.

The developed model in this study shows the relationship between five variables – sustainable requirements, traditional requirements, sustainable perceived value, and sustainable perceived value, with purchase intention and attempts to increase the success of products with sustainable product design in the market. With this model, product designers can incorporate sustainable product requirements at an early phase of the product design. This research also provides methods that can assist with the segmentation of the green smartphone market by identifying the demographic differences in the model’s variables.

### 6.1. Theoretical implications

This study contributes to the literature on consumers’ purchasing intention and adds to previous research on the correlation between sustainable purchase intention and sustainable design requirements, and it also contributes to advancing and expanding the research on this topic in China. The essential contribution of this research is the incorporation of variables like sustainable requirements, traditional requirements, sustainable perceived value, and price sensitivity to propose an advanced sustainable purchasing model. It also aims at finding the significant correlation between the independent variables (sustainable requirements, traditional requirements sustainable perceived value, and price sensitivity), and the dependent variables (sustainable purchasing intention). In this way, insights can be gained into the positive effect of sustainable purchasing as well as on sustainable requirements and traditional requirements.

### 6.2. Managerial implications

The findings show that demographic variables have a significant influence on sustainable smartphone purchasing intention. This finding might enable smartphone companies to segment the green market and develop effective sustainable marketing strategies. It helps companies to gain a deeper understanding of consumers who have a concern about the environment and sustainable design and develops more effective target customer marketing strategies. As well as the sustainable perceived value factors (social value, functional and emotional value) which could help companies decrease the possibility of new products failing in the market and increase opportunities for them to upgrade the features of new smartphones.

The proposed model in this research verified the positive impact of sustainable and traditional requirements on purchase intention. This could provide useful information for smartphone producers to design eco-friendly products and new marketing strategies that improve the requirements of the design and give it more attention than increasing their productivity.

And also the results of this research show a negative influence of price sensitivity on sustainable purchase intention so companies that produce smartphones are recommended to shift customers’ attention and consideration toward the price of the smartphones with sustainable requirements by highlighting the importance and the value of sustainable design requirements such as safety, lower electromagnetic radiation, energy saving, easy maintenance, durability, information, and data security, etc.

### 6.3. Limitations and future research opportunities

There are two limitations to this research. First, we cannot generalize the results. The relationship between sustainable requirements, traditional requirements, sustainable perceived value, price sensitivity, and purchasing intention is experimentally tested on smartphones and tablets in China. The respondents were from China, and the results could be different in other countries due to variations in the market, culture, and living standards. However, it is important to explore if the findings and conclusions can be applied to certain different other sustainable product designs and regions.

Future research could be conducted on different product designs and other regions. Second, this research was to investigate the relationship between sustainable requirements, traditional requirements, and sustainable purchasing intention, and only focus on the mediating effect of sustainable perceived value and the moderating effect of price sensitivity while excluding other possible mediating and moderating variables like brand loyalty and environmental consciousness. in future research, we will further refine the model and take moderating variables like brand loyalty and environmental consciousness into consideration in order to determine the relationship between sustainable requirements, traditional requirements, and purchase intention. Future studies could enhance this research results too by adding more factors such as brand and quality values.

## Data availability statement

The raw data supporting the conclusions of this article will be made available by the authors, without undue reservation.

## Ethics statement

Ethical review and approval were not required for the study on human participants in accordance with the local legislation and institutional requirements. Written informed consent for participation was not required for this study in accordance with the national legislation and the institutional requirements.

## Author contributions

LH wrote the paper, collected the data, performed the analysis, conceived and designed the analysis, and contributed to data and analysis tools. LD reviewed the article. All authors contributed to the article and approved the submitted version.

## Conflict of interest

The authors declare that the research was conducted in the absence of any commercial or financial relationships that could be construed as a potential conflict of interest.

## Publisher’s note

All claims expressed in this article are solely those of the authors and do not necessarily represent those of their affiliated organizations, or those of the publisher, the editors and the reviewers. Any product that may be evaluated in this article, or claim that may be made by its manufacturer, is not guaranteed or endorsed by the publisher.

## Figures and Tables

**Table:** 

		Items
**Sustainable requirements**		1. Smartphones must be recyclable. 2. Deference towards smartphones that are energy efficient. 3. Smartphones should be safe to use. 4. Smartphones should be easy to maintain. 5. There should be no toxic emissions or leakage risks. 6. Waste reduction is a priority. 7. Durable smartphones have more utility. 8. Preference for smartphones with lower SAR values. 9. Information and data security are extremely important.
**Traditional requirements**	Structure design requirements	1. Preference for smartphones with screens with scratch protection and larger screen sizes. 2. The shape and color of smartphones should be pleasing to the eye. 3. Waterproofed smartphones are more preferable.
	Ease-to-use requirements	1. Preference for a smartphone that is ergonomic, and thus easy to access. 2. Disassembly should be achievable, leading to easy repair.
	Performance requirements	1. The screen resolution and quality of smartphones should be high. 2. Smartphones with larger memory capacity are preferable. 3. Reliability of the device is of great importance. 4. The smartphones are built solidly and shockproof. 5. The processing speed of smartphones should be high. 6. The battery must be long-lasting. 7. The device should not cause interference with other devices. 8. The device should be 5G compatible.
**Sustainable perceived values**	Functional value	1. I prefer a smartphone with a variety of software apps for various uses 2. Get a significant value with the environmental features of smartphones.
	social value	1. I’m looking to purchase the smartphones that my relatives, friends, or work colleagues have chosen. 2. Smartphones that are more environmentally friendly can improve how people see me.
	emotional value	1. My smartphone makes me feel good. 2. I would like a smartphone since it is eco-friendly.
**Price sensitivity**		1. When it comes to purchasing a product, the price is the most essential consideration. 2. When purchasing a smartphone, the cheapest one is chosen. 3. All information and details are gathered on the price of the smartphone before purchasing.
**Purchase intention**		1. Intention to purchase a smartphone because it is concerned about the environment 2. Willingness to buy a smartphone is high.
